# A New Anatomical Classification for Tibialis Posterior Tendon Insertion and Its Clinical Implications: A Cadaveric Study

**DOI:** 10.3390/diagnostics11091619

**Published:** 2021-09-04

**Authors:** Jeong-Hyun Park, Digud Kim, Hyung-Wook Kwon, Mijeong Lee, Yu-Jin Choi, Kwang-Rak Park, Kwan Hyun Youn, Jaeho Cho

**Affiliations:** 1 Department of Anatomy & Cell Biology, School of Medicine, Kangwon National University, Chuncheon 24341, Korea; jhpark@kangwon.ac.kr (J.-H.P.); oe5235@naver.com (D.K.); kwenhw@naver.com (H.-W.K.); toff337@hanmail.net (M.L.); police5565@hanmail.net (Y.-J.C.); 2 Department of Anatomy, School of Medicine, Keimyung University, Daegu 42601, Korea; airboba@naver.com; 3 Division in Biomedical Art, Incheon Catholic University Graduate School, Incheon 21987, Korea; artanato@naver.com; 4 Department of Orthopaedic Surgery, Chuncheon Sacred Heart Hospital, Hallym University, Chuncheon 24253, Korea

**Keywords:** clinical anatomy, anatomical variation, anatomical implications, classification, tibialis posterior tendon, flatfoot, cadaveric study

## Abstract

The variations in the tibialis posterior tendon (TPT) could not be defined by previous classification; thus, this study used a larger-scale cadaver with the aim to classify the types of TPT insertion based on the combination of the number and location of TPT insertions. A total of 118 feet from adult formalin-fixed cadavers were dissected (68 males, 50 females). The morphological characteristics and measurements of TPT insertion were evaluated. Four types of TPT insertions were classified, wherein the most common type was type 4 (quadruple insertions, 78 feet, 66.1%), which was divided into four new subtypes that were not defined in the previous classification. The second most common type was type 3 (triple insertions, 25 feet, 21.2%) with three subtypes, including the new subtype. Type 2 was found in 13 feet (11%), and the rarest type was type 1 (2 feet, 1.7%), wherein the main tendon was only attached to the navicular bone and the medial cuneiform bone. We suggest high morphological variability of the TPT in relation to the insertion location, along with the possibility of significant differences according to race and gender. Moreover, this classification will help clinicians understand adult flatfoot deformity-related posterior tibial tendon dysfunction (PTTD).

## 1. Introduction

Adult acquired flat foot deformity (AAFD) is characterized by the collapse of the medial longitudinal arch, and its causes include posterior tibial tendon dysfunction (PTTD), rheumatoid arthritis, trauma (sequelae such as calcaneal fracture, tarsometatarsal joint injury, and ankle joint fracture), Charcot’s joint, and neuromuscular deficiency. In addition, rupture of the plantar fascia and the spring ligament complex as a static stabilizer that maintains the medial longitudinal arch has been suggested as the main cause. Among these causes, the most known clinical cause is PTTD, which is synonymous with AAFD in most references and textbooks [1,2,3,4,5,6].

The tibialis posterior tendon (TPT) acts as a primary dynamic stabilizer of the medial longitudinal arch and inverts, adducts, and plantar flexes the midfoot [1,7,8]. Because it is located more medially from the subtalar joint axis than other flexor tendons, the subtalar joint is inverted, and the foot becomes a rigid lever with adduction of the forefoot, locking the hindfoot and maintaining the medial longitudinal arch when the TPT contracts [9]. Thus, the loss of TPT function enables flatfoot deformity including hindfoot eversion, unlocking of the midtarsal joints, and plantarflexion of the talonavicular joint as well as forefoot abduction [10].

The tibialis posterior muscle originates from the interosseous membrane, while the posterior surface of the adjoining parts of the tibia, fibula, and muscle belly becomes the tibialis posterior tendon (TPT) in the distal third of the calf. The TPT is the largest and the most anterior of the medial ankle tendons, which acutely passes behind the medial malleolus [11,12]. The tendon inserts mainly into the navicular and medial cuneiform bones, but several additional insertions in the hindfoot and midfoot have been described [10,13,14,15,16,17,18]. In contrast to the anatomy associated with excursion, vascularity, and tendon sheaths in the TPT, the insertion anatomy has not been established in most studies to date but has been systematically classified into four main types according to the number of TPT insertions by Olewnik’s study [15] through dissection of 80 European cadavers in 2019. In addition, main types 3 and 4 were systematically classified into three subtypes (A–C) according to the location of additional insertions of the TPT. In our routine dissections of 10 Korean cadavers, however, new locations of additional insertion of the TPT that were not described at all in Olewnik’s classification were commonly observed.

Therefore, the current study was undertaken to ascertain the morphological characteristics of the tibialis posterior tendon (TPT) insertion using larger-scale dissection of Korean cadavers and to classify the types of TPT insertion based on the combination of the number and location of TPT insertions. The results of this study are expected to provide a reference to better understand the function of TPT through more detailed and extensive knowledge that will help distinguish different types of TPT insertion and further advance the epidemiology, diagnosis, and treatment of flatfoot deformity-related posterior tibial tendon dysfunction (PTTD).

## 2. Materials and Methods

This study was approved by the institutional ethics committee (Institutional Review Board number: NON2020-004).

All cadavers used in the present study were donated to the University of Medicine with consent for education and research. A total of 122 specimens were dissected. A total of 118 specimens were included in this study, while 4 specimens were excluded due to abnormal signs of trauma, surgery, obvious deformities, or pathologic lesions.

Of the 118 specimens dissected from adult formalin-fixed cadavers, 68 (55.7%) were from males and 50 (44.3%) were from females. The mean age of the donors at death was 74.5 (range, 44–100) years (Table 1).

### 2.1. Dissection

The cadavers fixed in formalin were placed in the prone position. The lower limb was then fixed in the prone position, so that the posterior tibial tendon was clearly visible. Dissection started from the area of the sole of the foot to the posterior aspect of the calf. After removal of the skin and superficial fascia, the soft tissue was dissected to expose the posterior tibial muscle. While confirming that the posterior tibial muscle courses to the ankle, the medial malleolus was exposed.

Following this, the flexor retinaculum was removed to check the course of the TPT from the ankle toward the sole of the foot. The plantar aponeurosis and the three layers of the sole were sequentially dissected from the sole of the foot. Thereafter, the fusion between the TPT and the third layer of the plantar muscle and tendon was carefully checked. Additionally, the location of bony insertions of the TPT was checked, as the tendons were dissected precisely to the bone attachments themselves.

### 2.2. Assessment of Morphological Characteristics of the TPT Insertion

Based on the criteria used in Olewnik’s classification [15], we classified the main types according to the number of TPT insertions. In addition, each was classified into subtypes (A–C) according to the anatomical structures and locations of bony insertion where the accessory bands of TPT were distally attached. If the new subtype found in this study was not included in the Olewnik’s classification, it was to be classified as subtype “K”. Considering the racial differences in the variation of the TPT insertion, the initial “K”, which means the variation found in the Korean cadavers, was used. Each independent researcher repeatedly assessed the morphology of TPT insertion twice. Identical consensus assessments from the two researchers were used as data for each specimen.

In addition, the width of the insertion of the main TPT was measured using the method of Swanton et al. [16]. The tendon width was measured at the point where the tendon began to widen before attaching the bone, 2 cm proximal to the insertion of the TPT to the navicular bone and the medial cuneiform bone. An electronic digital caliper was used for all measurements. Each measurement was performed with an accuracy of up to 0.1 mm. For all measurements, two researchers independently measured the width, and one researcher measured each measurement, and the average was adopted as the measurement value.

### 2.3. Statistical Analysis

The chi-square test was used to assess the distribution of TPT insertion between sex and the existing classification. All statistical analyses were performed using SPSS software (version 25.0; SPSS, Chicago, IL, USA) and a *p* value less than 0.05.

## 3. Results

The tibialis posterior muscle and tendon were present in all the specimens. It can be classified into four main types based on the morphology of the distal insertion.

**Figure 1:**
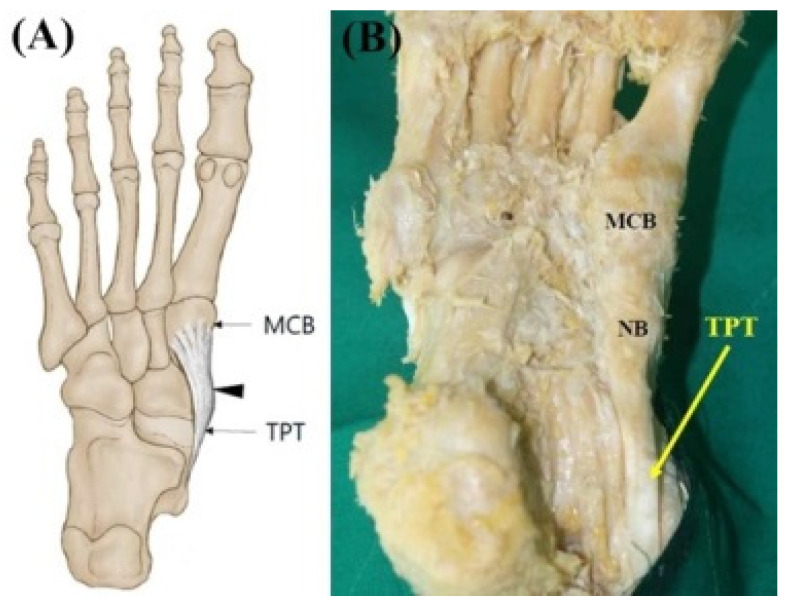
(**A**) Schematic drawing of the Type 1 tibialis posterior tendon insertion. Black-colored arrow indicates TPT inserts to the navicular bone. (**B**) Photo of the Type 1 tibialis posterior tendon insertion. The main tendon inserts to the navicular bone and the medial cuneiform bone (yellow-colored arrow). TPT, tibialis posterior tendon; NB, navicular bone; MCB, medial cuneiform bone.

Type 1 (single insertion) consisted of a single tendon. The main tendon only inserts into the navicular bone and medial cuneiform bone. This type was observed in two feet (1.7%) (Figure 1).

**Figure 2:**
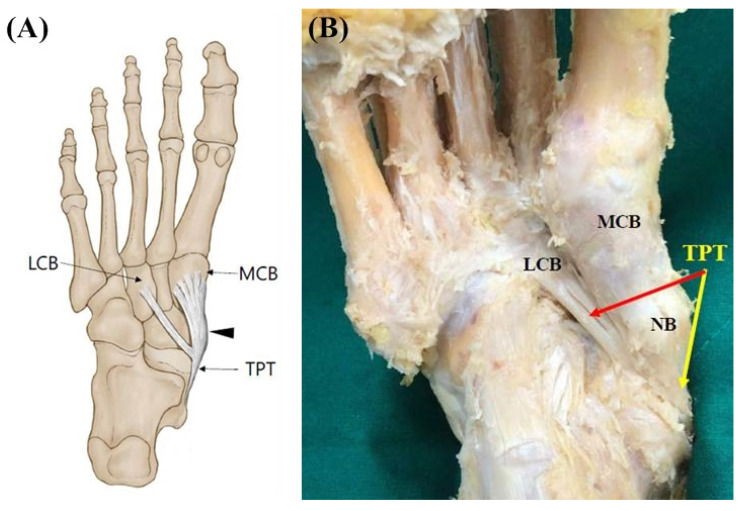
(**A**) Schematic drawing of the Type 2 tibialis posterior tendon insertion. Black-colored arrow indicates TPT inserts to the navicular bone. (**B**) Photo of the Type 2 tibialis posterior tendon insertion. The main tendon inserts to the navicular bone and the medial cuneiform bone (yellow-colored arrow). The second accessory tendon inserts to the lateral cuneiform bone (red-colored arrow). TPT, tibialis posterior tendon; NB, navicular bone; LCB, lateral cuneiform bone; MCB, medial cuneiform bone.

Type 2 (double insertions) consists of two tendons. The main tendon inserts, as in Type 1, and an accessory tendon inserts into the lateral cuneiform bone. This type was found in 13 feet (11.0%) (Figure 2).

**Figure 3:**
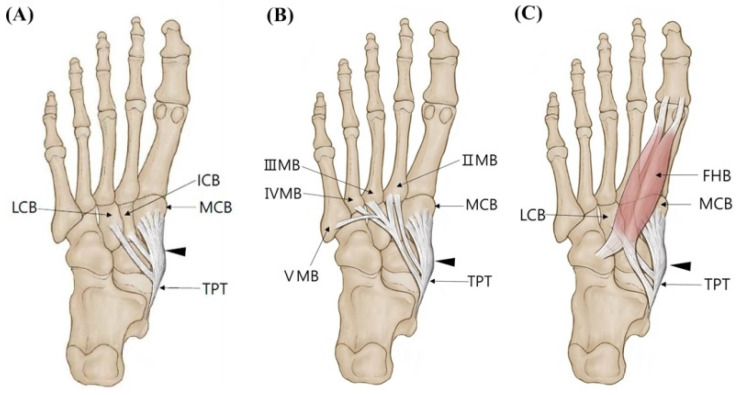
Schematic drawing of the Type 3 tibialis posterior tendon insertion. Black-colored arrow indicates TPT inserts to the navicular bone. (**A**) Type 3B. (**B**) Type 3C. (**C**) Type 3K. TPT, tibialis posterior tendon; MCB, medial cuneiform bone; LCB, lateral cuneiform bone; ICB, intermediate cuneiform bone; II, III, IV, V MB, second, third, fourth, fifth metatarsal bones; FHB, flexor hallucis brevis.

**Figure 4:**
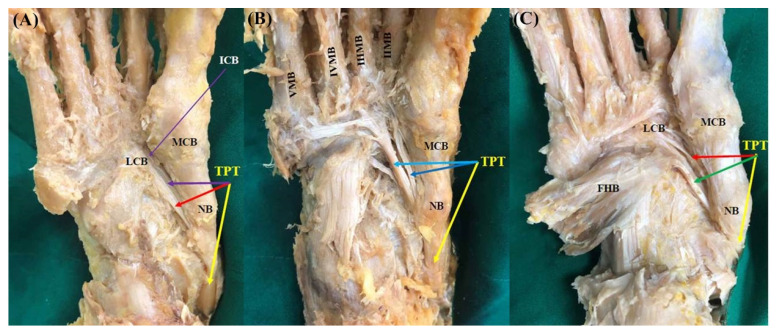
Photo of the Type 3 tibialis posterior tendon insertion. Yellow-colored arrow indicates main tendon inserts to the navicular bone and the medial cuneiform bone. (**A**) Type 3B. The first accessory tendon inserts to the lateral cuneiform bone (red-colored arrow). The second accessory tendon inserts to the intermediate cuneiform bone (purple-colored arrow). (**B**) Type 3C. The first accessory tendon inserts to the second metatarsal bone (blue-colored arrow). The second accessory tendon inserts to the second, third, fourth, fifth metatarsal bones (sky-blue-colored arrow). (**C**) Type 3K. The first accessory tendon inserts to the lateral cuneiform bone (red-colored arrow). The second accessory tendon inserts to medial arm of the fibrotendinous origin of to the flexor hallucis brevis muscle (green-colored arrow). TPT, tibialis posterior tendon; NB, navicular bone; MCB, medial cuneiform bone; LCB, lateral cuneiform bone; ICB, intermediate cuneiform bone; II, III, IV, V MB, second, third, fourth, fifth metatarsal bones; FHB, flexor hallucis brevis.

Type 3 (triple insertions) consists of three tendons, and the main tendon inserts as in Type 1; however, the following subtypes were divided according to the different insertion of the accessory tendons. Type 3 was observed in 25 feet (21.2%). In subtype 3A described in Olewnik’s classification, the first accessory tendon inserts together with the main tendon to the medial cuneiform bone, and the second accessory tendon inserts into the lateral cuneiform bone. However, this subtype was not observed in the present study. In subtype 3B observed in 5 feet (4.3%), the first accessory tendon was inserted into the intermediate cuneiform bone, and the second accessory tendon was inserted into the lateral cuneiform bone (Figure 3A and Figure 4A). In subtype 3C observed in nine feet (7.6%), the first accessory tendon posteriorly inserts to the II metatarsal bone, whereas the second accessory tendon anteriorly inserts to the second, third, fourth, or fifth metatarsal bones (Figure 3B and Figure 4B). In our study, a new subtype not mentioned in Olewnik’s classification was found in 11 feet (9.3%). In this subtype named “3K”, the first accessory tendon inserts to the lateral cuneiform bone, and the second accessory tendon inserts to medial arm of the fibrotendinous origin of the flexor hallucis brevis muscle (Figure 3C and Figure 4C).

**Figure 5:**
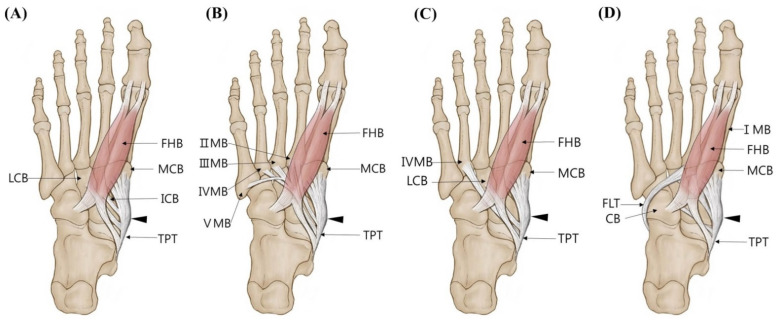
Schematic drawing of the Type 4 tibialis posterior tendon insertion. Black-colored arrow indicates TPT inserts to the navicular bone. (**A**) Type 4K-1. (**B**) Type 4K-2. (**C**) Type 4K-3. (**D**) 4K-4. TPT, tibialis posterior tendon; MCB, medial cuneiform bone; ICB, intermediate cuneiform bone; LCB, lateral cuneiform bone; FHB, flexor hallucis brevis; I, II, III, IV, V MB, first, second, third, fourth, fifth metatarsal bones; FHB, flexor hallucis brevis; CB, cuboid bone; FLT, fibularis longus tendon.

**Figure 6:**
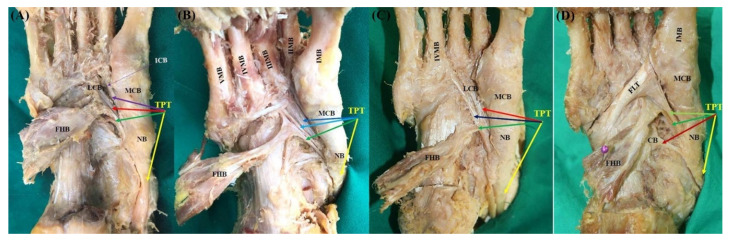
Photo of the Type 4 tibialis posterior tendon insertion. Yellow-colored arrow indicates main tendon inserts to the navicular bone and the medial cuneiform bone. (**A**) Type 4K-1. The first accessory tendon inserts to the intermediate cuneiform bone (purple-colored arrow). The second accessory tendon inserts to the lateral cuneiform bone (red-colored arrow). The third accessory tendon inserts to medial arm of the fibrotendinous origin of the flexor hallucis brevis muscle (green-colored arrow). (**B**) Type 4K-2. The first accessory tendon inserts to the second metatarsal bone (blue-colored arrow). The second accessory tendon inserts to the second, third, fourth, fifth metatarsal bones (sky-blue-colored arrow). The third accessory tendon inserts to medial arm of the fibrotendinous origin of the flexor hallucis brevis muscle (green-colored arrow). (**C**) Type 4K-3. The first accessory tendon inserts to the lateral cuneiform bone (red-colored arrow). The second accessory tendon inserts to the fourth metatarsal bone (navy-colored arrow). The third accessory tendon inserts to medial arm of the fibrotendinous origin of the flexor hallucis brevis muscle (green-colored arrow). (**D**) Type 4K-4. The first accessory tendon is conjoined with the fibularis longus tendon and insert together to the first metatarsal bone (yellow-green-colored arrow). The second accessory tendon inserts to the cuboid bone (red-colored arrow). The third accessory tendon inserts to medial arm of the fibrotendinous origin of the flexor hallucis brevis muscle (green-colored arrow). TPT, tibialis posterior tendon; NB, navicular bone; MCB, medial cuneiform bone; LCB, lateral cuneiform bone; ICB, intermediate cuneiform bone; II, III, IV, V MB, second, third, fourth, fifth metatarsal bones; FHB, flexor hallucis brevis.

Type 4 (quadruple insertions) consists of four tendons. The main tendon inserts as in Type 1, with the following subtypes divided according to the insertion of the accessory tendons. Type 4 was observed in 78 (66.1%) feet. It is noteworthy that three subtypes (4A, 4B, and 4C) described in the Olewnik classification were not found at all. In contrast, four new subtypes were identified in our study. In the subtype named “4K-1”, the first accessory tendon inserts to the intermediate cuneiform bone, the second accessory tendon inserts to the lateral cuneiform bone, and the third accessory tendon inserts to the medial arm of the fibrotendinous origin of the flexor hallucis brevis muscle (Figure 5A and Figure 6A). Subtype 4K-1 was observed in 25 feet (21.2%). In the subtype named “4K-2”, the first accessory tendon inserts to the second metatarsal bone, the second accessory tendon inserts to the second, third, fourth, or fifth metatarsal bones, and the third accessory tendon inserts to the medial arm of the fibrotendinous origin of the flexor hallucis brevis muscle (Figure 5B and Figure 6B). Subtype 4K-2 was observed in 38 feet (32.2%). In the subtype named “4K-3”, the first accessory tendon inserts into the lateral cuneiform bone, the second accessory tendon inserts to the fourth metatarsal bone, and the third accessory tendon inserts to the medial arm of the fibrotendinous origin of the flexor hallucis brevis muscle (Figure 5C and Figure 6C). Subtype 4K-3 was observed in 13 feet (11.0%). In the subtype named “4K-4”, the first accessory tendon inserts to the first metatarsal bone conjoined with the fibularis longus tendon, the second accessory tendon inserts to the cuboid bone, and the third accessory tendon inserts to the medial arm of the fibrotendinous origin of the flexor hallucis brevis muscle (Figure 5D and Figure 6D). Subtype 4K-4 was observed in two feet (1.7%).

The new classification of our study compared to Olewnik’s classification is summarized in Table 2. A significant difference in the distribution of types was observed between the two classifications (*p* < 0.001). This comparison is presented in Table 3. In addition, significant differences in the distribution of types was found between genders (*p* = 0.006), as presented in Table 4.

The measurement of the width of the insertion of the main tendon of the TPT is presented in Table 5. These results were measured in 109 feet, which was measurable in width regardless of the putrefaction of the cadavers. While the width of the navicular bone insertion was significantly wider in men than in women, the medial cuneiform bone insertion width did not differ significantly between genders.

## 4. Discussion

A clear understanding of the anatomical morphology of the tibialis posterior tendon (TPT) in relation to the insertion location is important for clinicians who encounter flatfoot deformity-related posterior tibial tendon dysfunction (PTTD). The first contribution of the current study is that the TPT insertion has high morphological variability and suggests the possibility of differences in the morphological variability of the TPT insertion by race, ethnicity, and gender. The second is to propose a new systematic classification of the TPT accessory tendons and their insertion type that can identify the type of TPT insertion that cannot be defined by the previous classification.

In the literature review, the main tendon of the TPT is mainly inserted into both the navicular bone and medial cuneiform, but the accessory tendons of the TPT have been described as attaching very diversely to the anatomical structures of the hindfoot and midfoot [10,13,14,15,16,17,18]. In addition to the navicular bone and medial cuneiform to which the main tendon inserts, the bones to which the accessory tendon can attach have been reported as intermediate cuneiform, lateral cuneiform, cuboid, calcaneus, and bases of all metatarsal bones. In particular, the insertion of TPT into the calcaneus was reported in 36 cases of 112 feet [17] and in 5 cases of 41 feet [18], but no calcaneal insertion of TPT was found in any systematic classification suggested by Olewnik’s study and the current study. In addition, soft tissues other than bones to which accessory tendons of TPT can be attached to have been reported, such as the plantar calcaneonavicular ligament, flexor hallucis brevis muscle, abductor hallucis muscle, and fibularis longus tendon [10,13,14,15,16,17]. Although Bloome et al. [13] presented five cases in which TPT was distally attached to the abductor hallucis muscle in a study of 11 feet, no case was observed in the systematic classification suggested by Olewnik’s study and the current study as well as in any other literature. Therefore, it is clear that the morphology of TPT insertion is highly variable. For this reason, the importance of the current study is to suggest a new classification by supplementing the subtypes not presented in the first systematic classification proposed by Olewnik through dissection of the larger samples.

A noteworthy point compared with the classification by Olewnik is that the distribution of types in the current study is significantly different. In Olewnik’s classification conducted in European populations, the number of TPT insertions tended to be rather small, and it was confirmed that TPT in more than two-thirds in our classification conducted in Korean populations consisted of one main tendon and three accessory tendons. This clearly suggests a difference in the anatomical morphology of TPT insertion by race or ethnicity. Although few studies on the incidence of adult flatfoot have been reported, a study reporting the frequency of adult flatfoot among Americans showed a significant difference in the occurrence of flatfoot based on the race of the individual [19]. Further, in this study, the distribution of the number of TPT insertions showed a significant difference by gender. Although there are no reports on the association between sex and flatfoot diagnosis in Korea, it is known from previously reported studies that female sex is more strongly associated with flatfoot diagnosis than age or BMI [20,21]. Therefore, it is considered that the morphological difference of the TPT insertion between races and gender may have some influence on the difference in the frequency of adult flatfoot.

Traditionally, posterior tibial tendon dysfunction (PTTD) has been understood as the main cause of adult acquired flatfoot deformity (AAFD) [1,2,3,4,5,6]. Therefore, surgical treatment has prioritized procedures that augment or replace TPT, and flexor digitorum longus (FDL) tendon transfer to the navicular bone has been most commonly used. However, recent studies have raised considerable uncertainty regarding the ability to correct deformities using FDL tendon transfer [22,23,24,25]. Additional procedures with FDL tendon transfer are commonly required at the distal site from the navicular bone to increase medial arch instability [26,27]. In all cadavers in this study, the main tendon of the TPT was inserted into both the navicular bone and medial cuneiform, and the width of the TPT attached to each of the two bones was almost equal to 1:1. It is generally reported that the flexor hallucis brevis has a fibrotendinous origin and originates from the lateral cuneiform and the cuboid, with some fibers (medial arm of the Y-shaped fibrotendinous origin) inserting the metatarsal component of the TPT [28,29]. In addition, in this study of Korean populations, it was found that the accessory tendon of the TPT was associated with FHB muscle in more than two-thirds of the feet. These anatomical results are thought to explain the uncertainty about the corrective ability for medial arch collapse when the FDL tendon is transferred only to the navicular bone. Recently, satisfactory outcomes have been reported by transferring the flexor hallucis longus (FHL) tendon to the base of the first metatarsal bone, which is the distal portion of the medial cuneiform in order to secure the stability of the entire medial longitudinal arch of the foot [30]. Therefore, the anatomical classification and morphological characteristics of TPT derived from our study may provide surgeons with basic knowledge and rationale for establishing the concept of stabilizing the medial column in tendon transfer procedures for surgical treatment of AAFD.

In addition, the classification system originally developed for posterior tibial tendon deficiency (PTTD) has been clinically applied to AAFD, and each stage from I to IV requires a different approach to management [31,32]. Although stage II PTTD patients often benefit from reconstruction of the deformity, unlike stage I patients, who are generally managed non-surgically, and those in stage III with rigid deformities that are better treated with arthrodesis, these patients are most controversial in terms of the choice of surgical procedures [33]. Over the past two decades, flexible AAFD has been commonly treated with a combination of FDL tendon transfer, various osteotomy (medial calcaneal osteotomy, lateral column lengthening osteotomy, Cotton osteotomy), heel cord lengthening, and spring ligament reconstruction [26]. Our new systematic classification based on the number and anatomical location of TPT insertions may distinguish how much TPT affects the degree of flatfoot deformity, so it is clinically important to determine the combination of various surgical procedures, especially in the surgical planning for stage II PTTD.

This study was limited by the use of fixed cadavers to evaluate the morphological characteristics of the tendons. Regarding post-mortem changes, there may be differences in the measurements taken from a live person and those from a cadaver. Furthermore, the cadavers were limited to those of elderly individuals (mean age 74.5 years), and the presence of tendon pathology may not be fully certain, because the corpse specimens donated for research do not provide the past medical history. In addition, clinical tendon pathology, such as PTTD, cannot be distinguished from fixed cadavers.

## 5. Conclusions

The present study suggests high morphological variability of the tibialis posterior tendon (TPT) in relation to the insertion location, along with the possibility of differences according to race and gender. In addition, a new systematic classification capable of identifying highly variable TPT insertion types will help clinicians to understand the stabilization of the medial longitudinal arch, especially in the treatment of adult flatfoot deformity-related posterior tibial tendon dysfunction (PTTD).

## Figures and Tables

**Table 1:** Gender and age distribution of Korean cadavers. (*n* = 118).

Age	Male	Female	Total
41~50	4	0	4
51~60	8	2	10
61~70	16	9	25
71~80	20	12	32
81~90	20	20	40
91~100	0	7	7
Total	68	50	118

The data are presented as number.

**Table 2:** Summary of classification for the insertion of the tibialis posterior tendon in current study compared to the previous study.

Classification	Type	Subtype	Main Tendon	Accessory Tendons	*n* (%)
Olewnik’s study [15]	1		NB, MCB		13 (16.25)
	2		NB, MCB	LCB	18 (22.5)
	3	A	NB, MCB	MCB, LCB	10 (12.5)
		B	NB, MCB	ICB, LCB	20 (25.0)
		C	NB, MCB	II MB, II~V MB	5 (6.25)
	4	A	NB, MCB	PCCL, CB, FHB	7 (8.8)
		B	NB, MCB	LCB, II MB, FHB	3 (3.7)
		C	NB, MCB	LCB, ICB, I MB + FLT	4 (5.0)
	Sum				80 (100)
Current study	1		NB, MCB		2 (1.7)
	2		NB, MCB	LCB	13 (11.0)
	3	B	NB, MCB	ICB, LCB	5 (4.3)
		C	NB, MCB	II MB, II~V MB	9 (7.6)
		K	NB, MCB	LCB, FHB	11 (9.3)
	4	K-1	NB, MCB	ICB, LCB, FHB	25 (21.2)
		K-2	NB, MCB	II MB, II~V MB, FHB	38 (32.2)
		K-3	NB, MCB	LCB, IV MB, FHB	13 (11.0)
		K-4	NB, MCB	FLT+ I MB, CB, FHB	2 (1.7)
	Sum				118 (100)

The data are presented as number (percent). NB, navicular bone; MCB, medial cuneiform bone; LCB, lateral cuneiform bone; ICB, intermediate cuneiform bone; I, II, III, IV, V MB, first, second, third, fourth, fifth metatarsal bones; FHB, flexor hallucis brevis; CB, cuboid bone; FLT, fibularis longus tendon; PCCL, plantar calcaneocuboid ligament; CB, cuboid bone; FLT, fibularis longus tendon.

**Table 3:** Comparison of distribution of types between two classifications.

		Olewnik’s Study [15]	Current Study	*p* Value
Type	1	13 (16.25)	2 (1.7)	<0.001
	2	18 (22.5)	13 (11.0)	
	3	35 (43.75)	25 (21.2)	
	4	14 (17.5)	78 (66.1)	
Sum		80 (100)	118 (100)	

The data are presented as number (percent).

**Table 4:** Distribution of types according to the gender (*n* = 118).

		Gender		*p* Value
		Male	Female	
Type	1	0 (0.0)	2 (4.0)	0.006
	2	11 (16.2)	2 (4.0)	
	3	9 (13.2)	16 (32.0)	
	4	48 (70.6)	30 (60.0)	
Sum		68 (100.0)	50 (100.0)	

The data are presented as number(percent).

**Table 5:** The width of the insertion of the main tendon of the tibialis posterior (*n* = 109).

Width	Male	Female	*p* Value
Insertion to navicular bone	11.60 ± 1.74	10.82 ± 1.20	0.007
Insertion to medial cuneiform bone	12.47 ± 2.47	11.60 ± 2.16	0.057

The data (in mm) are presented as mean ± standard deviation.

## Data Availability

The data used to support the findings of this study are available from the corresponding author upon request.
